# Comparison of the Internal Dynamics of Metalloproteases Provides New Insights on Their Function and Evolution

**DOI:** 10.1371/journal.pone.0138118

**Published:** 2015-09-23

**Authors:** Henrique F. Carvalho, Ana C. A. Roque, Olga Iranzo, Ricardo J. F. Branco

**Affiliations:** 1 UCIBIO-REQUIMTE, Department of Chemistry, Faculty of Science and Technology, Universidade NOVA de Lisboa, 2829-516 Caparica, Portugal; 2 Instituto de Tecnologia Química e Biológica António Xavier, Universidade Nova de Lisboa, Av. da República, 2780–157 Oeiras, Portugal; 3 Aix Marseille Université, Centrale Marseille, CNRS, iSm2 UMR 7313, 13397, Marseille, France; Oak Ridge National Laboratory, UNITED STATES

## Abstract

Metalloproteases have evolved in a vast number of biological systems, being one of the most diverse types of proteases and presenting a wide range of folds and catalytic metal ions. Given the increasing understanding of protein internal dynamics and its role in enzyme function, we are interested in assessing how the structural heterogeneity of metalloproteases translates into their dynamics. Therefore, the dynamical profile of the clan MA type protein thermolysin, derived from an Elastic Network Model of protein structure, was evaluated against those obtained from a set of experimental structures and molecular dynamics simulation trajectories. A close correspondence was obtained between modes derived from the coarse-grained model and the subspace of functionally-relevant motions observed experimentally, the later being shown to be encoded in the internal dynamics of the protein. This prompted the use of dynamics-based comparison methods that employ such coarse-grained models in a representative set of clan members, allowing for its quantitative description in terms of structural and dynamical variability. Although members show structural similarity, they nonetheless present distinct dynamical profiles, with no apparent correlation between structural and dynamical relatedness. However, previously unnoticed dynamical similarity was found between the relevant members Carboxypeptidase *Pfu*, Leishmanolysin, and *Botulinum* Neurotoxin Type A, despite sharing no structural similarity. Inspection of the respective alignments shows that dynamical similarity has a functional basis, namely the need for maintaining proper intermolecular interactions with the respective substrates. These results suggest that distinct selective pressure mechanisms act on metalloproteases at structural and dynamical levels through the course of their evolution. This work shows how new insights on metalloprotease function and evolution can be assessed with comparison schemes that incorporate information on protein dynamics. The integration of these newly developed tools, if applied to other protein families, can lead to more accurate and descriptive protein classification systems.

## Introduction

Proteases are a vast class of enzymes found in all kingdoms of life that participate in a wide range of biological processes [1]. They present different catalytic chemistries, structures, specificities, oligomeric states, and are grouped into distinct families and clans according to different classification schemes. Examples are the MEROPS [2], SCOP [3], and CATH databases [4], which use a combination of sequence- and structure-based methods for grouping distinct proteins. The need for a better understanding of their function has also led to the search of other common features shared between a wide range of known class members [5,6]; perhaps the most pervasive similarity was identified by Tyndall *et al*., where it was observed an almost universal binding of the Aspartic, Serine, Cysteine, and Metallo- proteases to the extended β-strand conformations of substrates, products, and inhibitors of peptidic and non-peptidic origin [5]. Nonetheless, there is still the need for a better characterization of the multiple factors governing protease function and evolution. We therefore tested if the employment of novel protein comparison tools can, when combined with conventional comparison methods, help in the search of additional features shared between distinct protease members.

Protein internal dynamics plays an important role on enzyme function, since it encompasses the space of catalytically-relevant structural changes occurring in a given fold during the reaction path [7–13]. These structural changes span a broad range of time-scales and magnitudes; from harmonic vibrations of bonds and angles occurring at the femtosecond time-scale, to global conformational fluctuations of large domains at the microsecond time-scale, some of them associated with substrate binding and product release [14–18]. Therefore, understanding the relation between protein internal dynamics and its structural and functional features is a challenging task, since it depends not only on the analysis of protein dynamics at different time scales, but also on their functionally-relevant molecular states (*e*.*g*. bound *vs* unbound state).

There is an ongoing debate on how protein internal dynamics is subject to evolutionary selection due to its functional significance and how it is related to sequence and structure evolution [19,20]. It has been shown that internal dynamics and backbone flexibility are conserved in homologous proteins [6,21–27]. Specifically, it has been observed that: *i)* low-frequency, large-amplitude normal modes tend to be evolutionarily conserved [24,28] and; *ii)* there is a significant correspondence between low-energy modes determined for superfamily structural cores and the modes of structural variance observed within protein superfamilies [29,30]. These findings support the notion that conservation of protein dynamics is subject to evolutionary selection and were based on the observation that ligand binding can be described in many cases by few low-energy normal modes [31,32]. However, similarities of low-energy modes observed between non-homologous proteins with the same architecture and even between unrelated proteins [26,33], together with the observation that low-energy modes are more robust to random mutations suggests that protein dynamics may not always be subject to evolutionary selection [34–36].

Additional insights have been provided by phylogenetic studies addressing the evolution of normal modes. It was shown that changes in protein dynamics are associated with functional divergence in enzymatic families and that non-homologous enzymes that perform similar functions also share similar motions related to catalysis [37,38]. Protein dynamics has also been found to diverge between structurally related proteins at functionally important sites [39,40], and this divergence has been argued to be dependent on other functional requirements, such as intracellular localization [41]. Finally, protein dynamics has also been associated with the evolution of new enzymatic functions and on the promiscuity of enzymes [42–45]. These findings suggest that protein dynamics plays an important role in the function and evolution of enzymes, although the extent to which evolution has selected for this particular trait still remains unclear.

The development of dynamics-based comparison methods has been crucial to the above mentioned studies and has provided insights that may not be detectable from sequence or structure comparison methods alone [27,40,46–52]. These methods rely on Essential Dynamics Analysis (EDA), to retrieve the collective motions of protein structures, which are typically obtained from Molecular Dynamics (MD) simulations or alternatively by simplified, coarse-grained representations of protein structure, such as Elastic Network Models (ENM). The similarity between modes of two distinct structures can be compared based on different quantitative schemes and therefore new relations between proteins can be sought based on their dynamical properties [50].

In the search for common features shared between different types of proteases, Carnevale *et al*. carried out pairwise structure- and dynamics-based alignment of 17 representative protease structures with minimal mutual sequence identity and distinct folds [6]. In most cases the division into distinct folds was consistent with the division in clans of the MEROPS classification scheme, indicating that structures with different evolutionary origins adopt distinct folds. Nonetheless, significant structural similarities among proteases of different clans were identified, thus suggesting a convergent evolutionary process. Indeed, in pairs of structures showing higher structural similarity, the aligned segments in both structures consisted on regions close to the active-site, even for pairs from distinct clans (*i*.*e*. different catalytic chemistries). The authors proposed that a criterion for catalytic activity not dependent on chemical determinants could be at play, namely the dependence on specific and concerted protein motions related to function. A close correspondence of the internal dynamics between some pairs was identified, which consisted in rearrangements of active-site surroundings that lead to distortions of the substrate-accommodating pockets. Therefore, it was suggested that a “dynamical selection” process, operated by the necessity to interact with substrates in well-defined geometrical arrangements, may lead to convergence or conservation of the internal dynamics in proteases. Indeed, recent work reported by Micheletti further identified significant dynamical similarities between proteases from different clans with no detectable structural similarity [50]. It was therefore suggested that dynamics-based comparison methods could be useful in detecting functionally-related features shared by proteases that otherwise would remain elusive using only sequence- and structure-based methods.

Metalloproteases (MPs) are one of the most diverse types of proteases, presenting a wide range of folds and catalytic metal ions. They are divided in more than 40 families identified among all kingdoms of life. In the case of the MEROPS MA clan, where most of the known MPs are grouped by common ancestry, its members are characterized by a single catalytic zinc ion, a consensus HEXXH sequence motif and a common fold architecture. However, this structural conservation is not observed at the domain level since members from different families have distinct domain composition and topology. MPs are therefore attractive candidates to study the relationship between structure and dynamics within a protein clan. In this work, the suitability of employing coarse-grained methods to the study of MP internal dynamics was first made by comparison of ENM-derived internal dynamics profile of thermolysin to those obtained by Principal Component Analysis (PCA) of crystal structures and EDA of MD simulation trajectories. Subsequently, an analysis of pairwise structural- and dynamics-based alignments of a representative set of MPs from 13 families of the MA clan was performed. It was found for members of this clan that dynamical similarity does not appear to correlate with structural similarity. Interestingly, pairs having high dynamical similarity despite having no structural similarity were identified. Inspection of the produced alignments indicates that in these cases conservation of internal dynamics has a functional basis, namely to dictate proper interactions with the substrate. Our data show the suitability of using simple comparison schemes that incorporate information on dynamics to provide new insights on MPs function and evolution, unveiling their potential as tools to study the role of internal dynamics in protein evolution.

## Methods

### Internal Dynamics of Thermolysin

PCA of the structural set was made to obtain the respective Principal Components (PC), using the ProDy software [53,54]. The 3*N* x 3*N* covariance matrix was calculated over *n*, where *N* = 316 is the number of residues in thermolysin (represented by the respective C^α^ atom coordinates), and *n =* 112 is the number of thermolysin crystal structures (Uniprot ID: P00800) retrieved from the PDB [55], with corresponding IDs (Table A in S1 File). Structures were initially superposed to the unbound crystal structure (PDB ID: 1L3F) to obtain mean coordinates, then iteratively superposed until convergence to eliminate rigid-body translational and rotational differences.

MD simulation of thermolysin in the unbound form (PDB ID: 1L3F) was performed using GROMACS 4.6.1 simulation package with GPU acceleration [56–58], with the AMBER99SB-ILDN force field [59] (Sim1). The system was solvated with explicit Simple Point Charge (Extended) water model (SPC/E, [60]) and placed in a dodecahedral box, each edge at least 12 Å from the protein surface. The system was charge-neutralized by addition of two sodium counter-ions and minimized in two steps to remove atom clashes and bond contacts: first by a steepest descent minimization algorithm (2000 steps), followed by a conjugated gradient algorithm (1000 steps). The energy-minimized model was coupled to the V-rescale thermostat (300 K, coupling time constant 0.1 ps [61]) and Berendsen barostat (1 bar, coupling time-constant 0.6 ps [62]) and then equilibrated, where the force-constant of positional restraints for heavy-atoms was decreased from 1000 kJ/mol, 100 kJ/mol to 10 kJ/mol in three consecutive steps (100 ps). A production phase was finally run for 20 ns, with an integration step of 2 fs. Long-range electrostatics were treated with the Particle-Mesh Ewald algorithm and distance constraints between all H-bonds was enforced by the LINCS algorithm [63]. Although the employed force field does not appropriately represent the interactions between the catalytic zinc metal ion and the coordinated residues [64], this metal ion is not considered to have a structural role [65]. An additional non-biologically relevant zinc ion found at the active site of the crystallographic structure was removed. The protonation states of active site residues from the conserved HEXXH sequence motif were manually attributed, with H142 and H146 monoprotonated at the N^δ^ position and E143 and E146 not protonated, taking into account its specific pKa. A replicate of the MD simulation was carried out (Sim2), where all abovementioned simulation set-up parameters were kept unchanged. After removal of the global rotation and translation of the protein, simulation trajectories show a RMSD convergence after 1 ns of the production phase (Fig A in S2 File). Therefore, only the 1–20 ns interval of full trajectories were used for EDA with the ProDy software, with the 1 ns frame being used as the reference structure. EDA is based on PCA, with the difference that the respective PCs (termed here as ED modes) are calculated based on *n* = 9500 trajectory frames taken at intervals of 2 ps, considering only the *N* = 316 C^α^ atom coordinates.

The Anisotropic Network Model (ANM, [66–68]) of thermolysin in the unbound form (PDB ID: 1L3F) was calculated using the ProDy software. The C^α^ atom coordinates were used as node representations of each residue of the protein (*N* = 316) to build the respective 3*N*x3*N* Hessian matrix. Variations of the model included additional nodes, matching the coordinates of the catalytic zinc ion alone (*N* = 317) or the catalytic zinc ion and the four calcium ions (*N* = 321). The uniform force constant γ = 1 was used to calculate the overall potential of the system and the interaction cutoff distance r_c_ = 15 Å was used to generate the respective Kirchhoff matrix of inter-residue contacts.

The collectivity degree, *κ*, was used as a measure of the number of atoms significantly affected by a given PC, ED or NM mode [69]. This value varies from *κ* = 1 for modes describing global translations of the protein to *κ = N* ^-1^ if only one atom is affected (*N* = 316). The overlap, or correlation cosine, between two modes is given by the dot product of the respective eigenvectors after normalization, being equal to one if two modes are identical. The subspace overlap between two sets of modes is given by corresponding Root Mean Square Inner Product (RMSIP) value [70]. The respective overlap between the covariance matrices was also calculated, with a value of 1 if the two matrices are identical and of 0 when the respective subspaces are orthogonal [71].

### Selection of representative structures

A representative set of MP structures from distinct families belonging to the MA clan of the peptidase database MEROPS (release 9.9) was selected [2]. In MEROPS, members are grouped in families based on their sequence similarity. Families are further grouped in clans when there is detectable structural homology, implying common ancestry. For each of the 23 (out of 42) families that contain members with resolved structures in the PDB, a representative structure was selected based on the following criteria: *i)* unbound structure containing no inhibitor molecule, substrate or substrate analog molecule and; *ii)* the structure with highest resolution or with no mutations. Only unbound crystal structures were selected since they are assumed to represent the native conformation of the corresponding protein. Also, the degree and mode of conformational change upon substrate or inhibitor binding is not well characterized for most clan members as it is for thermolysin, and the effects associated with ligand heterogeneity can be ruled out. The resulting set is comprised of 13 structures belonging to distinct families. For each structure, the respective information from the SCOP and CATH databases was retrieved [3,4]. In MEROPS, MA clan members are grouped into the MA(E) and MA(M) subclans, commonly termed as gluzincins and metzincins, respectively. These two subclans are divided based on the nature of the third zinc ligand: in MA(E) a glutamate, 18–72 residues apart from the conserved HEXXH motif towards the C-terminal; in MA(M) an histidine or aspartate in the extended HEXXHXXGXXH/D motif [1]. All structures correspond to the monomeric form of the proteins and the majority is characterized by a two-domain peptidase unit, with a conserved N-terminal domain and a more variable C-terminal domain. The active site is generally located between these two domains. Despite these general features, the set is structurally heterogeneous, with proteins containing domains that differ at the class, architecture and topology level of CATH classification criteria. In most cases, MEROPS classification is coincident with SCOP classification at the family level, except for the SCOP family 55505 “Neurolysin-like” that combines MEROPS families M2, M3 and M32; and also family 55487 “Zinc Protease” that combines M7 and M35 families. In the case of families M10 and M12, which are divided into subfamilies due to deep sequence divergence between their members, a representative was selected for each subfamily. Representatives of the MA(E) subclan families M3, M4 and M27 are endopeptidases and M1, M2 and M32 are exopeptidases, while in the MA(M) subclan all representatives are endopeptidases.

### Structure-based alignments

Pairwise structural alignment of structures was performed with the DaliLite web-server for all 78 distinct pairs [72]. The embedded DALI algorithm identifies blocks of residues between two distinct structures that have similar inter-residue distances. Matching regions are evaluated based on a knowledge-based score and the produced alignment is the one maximizing this value for a variable number of distinct blocks. The statistical significance of the alignment is quantified in terms of a Z-Score that compares the obtained score with the one expected for a pair of structurally unrelated structures of the same size. A Z-Score greater than 2.0 is considered significant and was used as threshold value for a pair of structures to be considered as structurally similar [72]. In the MEROPS, a structure is grouped in a predefined clan if a Z-Score greater than 6.0 is obtained between the structure and at least one member of that clan. Therefore, not all pairs of representatives are expected to be structurally similar.

### Dynamics-based alignments

Dynamics-based alignment of all pairs used for structural alignments were performed using the ALADYN web server [51]. First, the implemented algorithm calculates the low-energy modes for each structure and then it detects regions of both proteins with similar dynamic profiles. Calculation of modes is based on the coarse-grained β-Gaussian ENM, where amino acids are represented by a two-centroid representation: C^α^ atom for the main chain and; C^β^ for the orientation of the side chains (except for Gly residues) [73,74]. This ENM has been shown to describe protein motions similarly to the employed ANM [75]. The dynamics-based alignment is made by rewarding superpositions of proteins regions that exhibit high overlap between the 10 first modes for each amino acid pair within the cutoff distance of 7 Å. This allows for the alignment of proteins with different sequences and size. Following the optimization of scoring function, the statistical significance of the resulting alignment is evaluated against a reference probability distribution of scores obtained from alignments of unrelated protein pairs, being expressed in terms of a P-value. Dynamics-based alignments of two structures with P-value smaller than 0.02 are considered statistically significant and was used as the cut-off value to consider two structures as dynamically similar [51].

## Results and Discussion

### Internal Dynamics of Thermolysin

**Fig 1:**
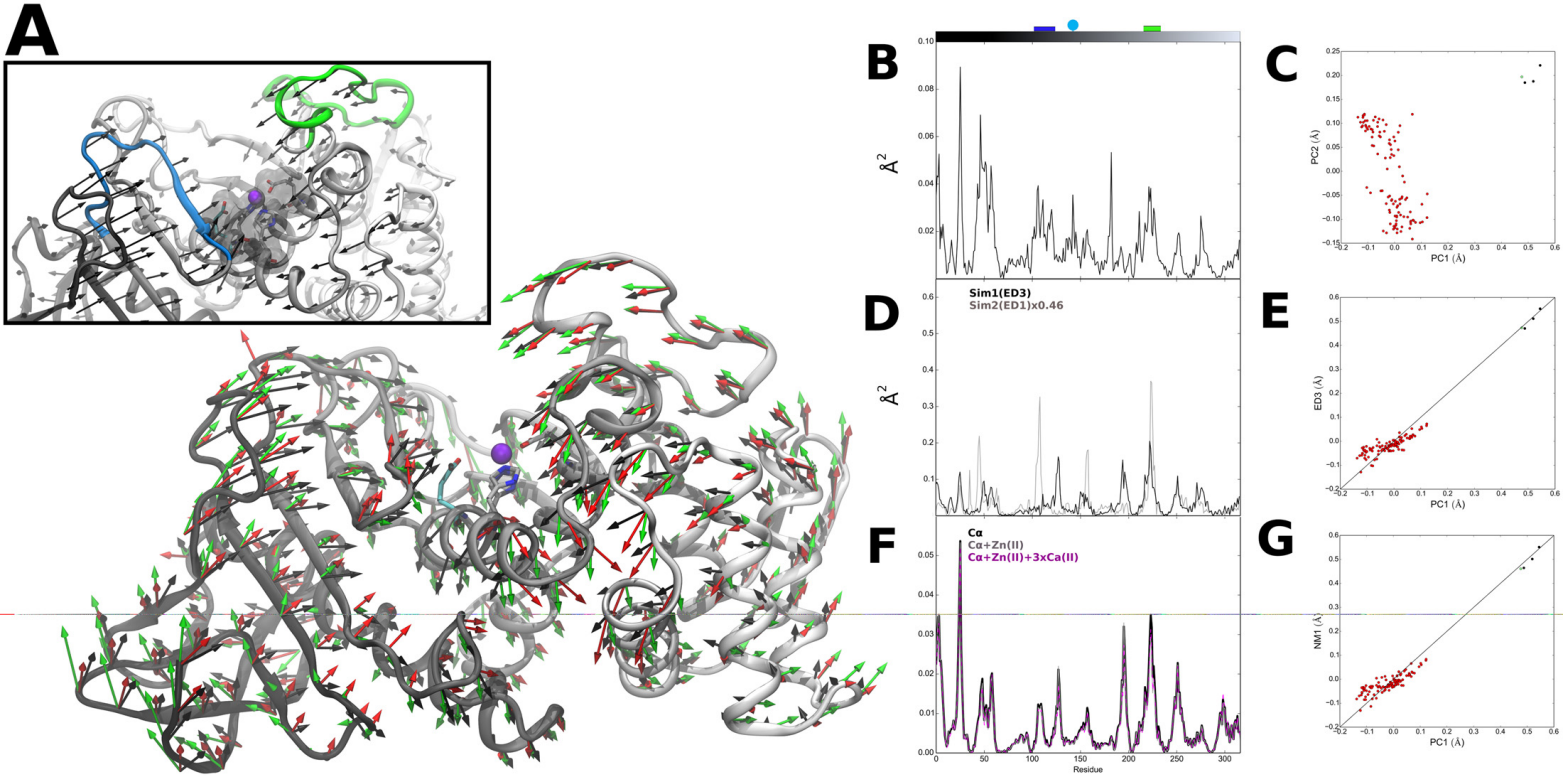
Thermolysin PC, ED and NM modes. (A) Visual representation of residue-level PC1 mode vectors (black, scale 2.05), NM1 mode vectors (green, scale 1.47) and ED3 mode vectors from Sim1 (red, scale 3.17). Inset: Details of active site region. Colored ribbons corresponding to the top of the active site pocket (residues 105–177 in blue and 210–230 in green). Active site residues H142, H146 and E166 in green and catalytic E143 in cyan sticks representation. (B) Square fluctuations as a function of residue index (1.316) obtained for PC1. Top bars correspond to residue coloring as presented in (A), with catalytic E143 represented as cyan circle. (C) Projection of thermolysin crystal structures along PC1 and PC2. Structures were grouped into bound (red) and unbound (black) groups; unbound reference structure (PDB ID: 1L3F) used in PCA, EDA and NM calculations (green). (D) Square fluctuations as a function of residue index obtained for ED3 from Sim1 and ED1 from Sim2. (E) Cross-projection of crystal structures along PC1 and ED3 from Sim1 (r = 0.94). (F) Square fluctuations as a function of residue index obtained for NM1 from calculated thermolysin ANM and respective variants. (G) Cross-projection of crystal structures along PC1 and NM1 (r = 0.95).

**Fig 2:**
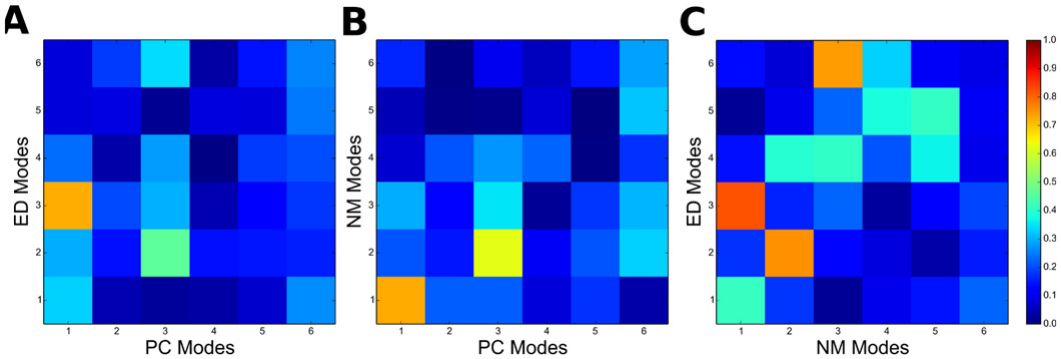
Comparison of the first six PC, ED and NM modes. (A) Overlap between PC and ED modes from Sim1; (B) Overlap between PC and NM modes and; (C) Overlap between NM and ED modes from Sim1.

Thermolysin, the MA clan type peptidase, is a 316 residue-long thermostable neutral MP from *Bacillus thermoproteolyticus* [1]. It presents endopeptidase activity towards peptide substrates, cleaving peptidic bonds preferentially close to aromatic residues. The active site contains a catalytic zinc ion bound to two histidines (H142 and H146) and one glutamate (E166) residue, and an additional catalytic glutamate (E143) residue [76,77]. It is located at the bottom of a pocket formed by the two protein domains: the N-terminal domain composed mostly of β-sheets containing the conserved HEXXH sequence motif with the corresponding H142, E143 and H146; the C-terminal domain composed mostly of α-helices, where E166 is located. Given our interest in analyzing and comparing the dynamical properties of MPs, we first characterized the dynamical features of thermolysin, particularly those that are functionally relevant. In order to do this, we used the ProDy software, and the type of results produced can be found in Figs 1 and 2.

Several studies of thermolysin have been made in the last decades, with multiple crystal structures obtained in different conditions available in the PDB. These structures provide snapshots of the motions undergone by the protein upon interaction with different molecules, including substrate-analogs and inhibitors, thus giving the opportunity to describe the conformational subspace related with its function [78–80]. For this purpose, we applied PCA to a set of 112 thermolysin crystal structures in order to characterize the collective modes of atomic displacements (see Methods section for details on PCA calculations) [78,81]. As shown in Table 1, a small set of PCs describes the majority of atomic positional variations occurring in the structural set, with the first six PCs (PC1-PC6) describing 80% of the total variance. PC1 alone accounts for 35% of the variance, with a cumulative displacement of < 5 Å. The respective structural variation for each residue along PC1 is shown in Fig 1A and 1B. Although these structural differences are small (0.60±0.08 Å), they are nonetheless highly collective, affecting approximately half of the atoms in the protein (0.43 ≤ κ ≤ 0.69). Higher fluctuations are seen for the N-terminal region (including the surface residues 42–62). Regions 105–117 and 210–230, which form the top of the active site pocket and contain residues involved in substrate binding [82], also exhibit high, anti-correlated variations (Fig 1A inset). Conversely, the pocket bottom region where the zinc binding residues are located shows relatively lower variation, with the exception of the catalytic residue E143. The structural fluctuations seen for this catalytic residue towards the pocket bottom reflects local accommodations of the structure to the presence of different ligand molecules in the active site [65, 83]. Projection of each structure onto the subspace spanned by PC1-PC2 is shown in Fig 1C. Two distinct clusters corresponding to the subsets of bound and unbound forms are obtained, as seen by the distribution of structures along the PC1 axis (P-value in Table 1). This indicates that the presence of molecules in the active site is associated with conformational changes in the protein that are effectively described by PC1. The variations described by PC1 point to an opening-closure movement of the active site pocket, *i*.*e*. an hinge-bending motion of N- and C-terminal domains with the vertex at the pocket bottom. Large scale, hinge-bending displacements were initially described for thermolysin and related neutral proteases [84]. The correspondence with hinge-bending motions was made by analysis of short MD trajectories and later confirmed by the reported unbound crystallographic structure of thermolysin [83,85]. Therein, the detected hinge-bending motions were related to transitions between “open” and “closed” conformations of the unbound and bound forms of the protein, respectively, indicating their functional role. Therefore, the current results show that PC1 alone can describe to a reasonable extent the functionally-relevant conformational changes of thermolysin, with motions in the positive direction along the PC1 axis describing an “opening” of the active site pocket and in the negative direction to its “closure”.

**Table 1:** Fraction of variance and collectivity of the first 6 PC, ED and NM modes obtained for thermolysin. The collectivity degree is expressed as κ and reflects the portion of atoms in the structure affected by a given mode.

PC modes			ED modes Sim1/Sim2			NM modes		
	Fraction of Variance (P-value)*	κ		Fraction of Variance	κ		Fraction of Variance	κ
PC1	0.35 (2.2x10^-16^)	0.69	ED1	0.23 /0.18	0.46/0.52	NM1	0.18	0.64
PC2	0.23 (5.9x10^-11^)	0.55	ED2	0.1/0.08	0.62/0.33	NM2	0.14	0.63
PC3	0.13 (1.8x10^-4^)	0.61	ED3	0.07/0.04	0.65/0.61	NM3	0.09	0.64
PC4	0.04 (8.0x10^-2^)	0.43	ED4	0.05/0.04	0.63/0.26	NM4	0.06	0.13
PC5	0.03 (1.0x10^-2^)	0.54	ED5	0.04/0.03	0.35/0.61	NM5	0.05	0.09
PC6	0.02 (4.1x10^-3^)	0.56	ED6	0.03/0.03	0.71/0.4	NM6	0.05	0.38

*Anderson-Darling normality test for the projection of structures along PC modes.

In order to test if the conformational changes described by the PCA are intrinsic, *i*.*e*. if they are encoded in the internal dynamics of thermolysin, we performed EDA on snapshot conformations from two replicates (Sim1 and Sim2) of a 20 ns MD simulation trajectory of the protein in its unbound state (see Methods section for details) [86,87]. A similar analysis has been previously reported for other proteases of different clans (with distinct catalytic chemistry) [79,88]. EDA is focused on the subspace of PCs, typically the top-ranking modes, that describe the majority of collective atomic motions along a simulation trajectory, and which are typically related to protein function. As indicated in Table 1, the first six EDA-derived PCs (ED1-ED6) have lower variance values than the corresponding structurally-derived PCs (PC1-PC6), with ED1-ED6 of Sim1 accounting for only 52% of the total variance (40% for Sim2). However, in terms of collectivity degrees, EDs and PCs are similar (0.35 ≤ κ ≤ 0.71 for Sim1 and 0.33 ≤ κ ≤ 0.61 for Sim2), reflecting the collective nature of motions described by ED1-ED6 modes. Although conformational changes related to ligand-binding typically occur at longer time-scales, EDA of MD simulations of a few ns generally provides a reasonable description of the full conformational space explored by the protein [70, 71]. During the total simulation time analyzed (19 ns after RMSD convergence), the sampled subspaces are convergent for each simulation. This is shown by comparing the covariance of residue fluctuations between two time intervals, the interval of the initial 11.4 ns and the full time interval. The overlap of covariance matrices obtained is 0.63 for Sim1 and 0.65 for Sim2. Moreover, the overlap between subspaces explored during the two time intervals yields a RMSIP of 0.93 for both Sim1 and Sim2. When comparing Sim1 against Sim2, the sampled conformational subspaces defined by the respective ED1-ED6 modes are similar, with a RMSIP of 0.79. However, the overlap between the respective covariance matrices is relatively low (0.38). These results indicate that the two simulations exhibit similar dynamical behavior during the analyzed time window, although the sampled conformational space explored are distinct, as it has been reported for other proteins [89,90].

While it remains uncertain if the protein explores a distinct conformational space on longer time scales, which would require simulation times from μs to ms or other methods more suitable to characterize the protein potential energy surface, the remaining analysis is focused on the correspondence between the results obtained from the current EDA with the experimentally-derived PCA of thermolysin. Therefore, in order to compare the obtained EDs with structurally-derived PCs, the overlap between each of the respective first six modes was calculated. As shown is Fig 2A, the highest value was obtained between modes ED3 and PC1 from Sim1, with an overlap of 0.72 (for Sim2 an overlap of 0.71 was obtained between ED1 and PC1). The large overlap between ED3 and PC1 from Sim1 translates in similar directions of residue fluctuations shown in Fig 1A and 1D, particularly in the region comprising the active site pocket. Further confirmation of the high similarity between these modes is obtained in Fig 1E, where cross-projection of crystal structures along PC1 and ED3 from Sim1 yields a distribution along the diagonal with a clear separation between bound and unbound subsets. Indeed, the large cumulative overlap of 0.85 between ED1-ED3 from Sim1 and PC1 (0.80 for ED1-ED3 and PC1 of Sim2) show that functionally-relevant conformational changes are effectively captured by the first three ED modes and therefore can be said to be encoded in the internal dynamics of the protein. However, there is low correspondence between subspaces defined by the respective modes, as given by the low RMSIP value of 0.49 for Sim1 and 0.46 for Sim2 between ED1-ED3 and PC1 (for ED1-ED10 and PC1-PC10 the RMSIP is 0.56 for Sim1 and 0.53 for Sim2) [70,91]. The obtained results show that a high conformational space is sampled during MD simulations, which includes a functionally-relevant subspace (particularly the ones described by ED3 from Sim1 or ED1 from Sim2) that is only explored upon interaction with the ligand (as described by PC1). This is in line with conformational selection models of protein function described elsewhere [80,92].

ENMs have been extensively evaluated against experimental and computational benchmarks [73,93–101]. They have also been used previously to describe functional aspects of MP internal dynamics, although the relation between collective motions of MPs and their function was only indirectly established [50]. Specifically, it was shown that Atrolysin E and other non-metallo proteases have similar dynamical profiles in the respective active site regions. However, regions close to metal-centers and active sites tend to exhibit similar and relatively restrained, dynamical profiles [102,103]. Since MPs contain a metal-center in their active site, the functional relevance of the respective ENM-predicted dynamical profiles should be more thoroughly addressed. For this purpose, we first tested the suitability of using coarse-grained ENMs in reproducing the internal dynamics of thermolysin. The ANM implemented in the ProDy software was employed to the unbound structure of thermolysin and the space of collective motions was characterized by Normal Mode Analysis (NMA) [54,93,104–106].

The ANM employs a C^α^-based node representation of protein structure. Given the presence of metal ions in clan MA members, the addition of node representations for metal ions was also evaluated for thermolysin, using a similar approach to the one made for another ENM [103]. Variations of thermolysin ANM were generated to represent the zinc ion found at the active site and also four additional calcium ions found at the N-terminal domain. While the later are crucial for the thermostability of the protein, the zinc ion is not considered to play a crucial structural role, since its metal-substituted forms present very similar tertiary structures [65]. Fig 1F shows that the respective residue square fluctuations profiles of the model variants produced are almost identical to the C^α^-only ANM (r > 0.995 for both variants), with only slight variations on fluctuation amplitudes in the 220–226 region, at the top of the active site pocket. The close similarity between the two variants indicates that the introduction of calcium nodes does not produce significant changes in the global dynamical profile of the protein, but the inclusion of the zinc node alone produces small local changes in the dynamic profile of the active site pocket. Since the calcium nodes are introduced at a highly clustered region of the network of inter-residue contacts, its topology is mostly unchanged. The introduction of the zinc node, on the other hand, leads to new network connections in the active site region, producing slight variations on its topology that nonetheless result in very similar dynamical profiles. Therefore, the inclusion of additional nodes for metal ion representation produces only local changes on the ANM-derived dynamical profile of thermolysin, the clan MA type protein.

The results above are in line with recent studies where the employment of coarse-grained models was evaluated for globular folds [21, 36], such as the one of thermolysin. It was found that the respective ENMs can effectively capture their essential dynamics [21] and that these models are robust to local perturbations [36]. Given the conserved tertiary structure of clan MA members, the accuracy of the respective ENMs in describing their internal dynamics is not expected to greatly increase with the inclusion of additional metal ion nodes. We therefore focused the remaining analysis on the results obtained for the C^α^-based ANM of thermolysin (*N* = 316), particularly on the subset of NMA-derived low-frequency NM modes (NM1-NM6).

As shown in Table 1, the variance of NM1-NM6 is significantly lower than the corresponding PCs and similar to ED modes, accounting for 57% of the total variance. In particular, NM1 accounts for only 18% of the total variance, similar to ED1 from Sim1 and Sim2. In terms of collectivity degrees, the three lowest-frequency modes (NM1-NM3) are highly collective (0.63 ≤ κ ≤ 0.64), in the range of PCs and EDs, and the remaining modes (NM4-NM6) have significantly lower values (0.09 ≤ κ ≤ 0.38). The overlap between NM modes and PCs is shown in Fig 2B. Remarkably, a large overlap of 0.71 is found between NM1 and PC1. Fig 1A shows the close correspondence between NM1 mode directions and PC1. Again, a high similarity of motions is observed in the region comprising the active site pocket. This similarity is also reflected in terms of the corresponding profiles of residue fluctuations in Fig 1F. Cross-projection of the structural set along NM1 and PC1 shown in Fig 1G further confirms the close correspondence between these modes. This indicates that the lowest-frequency mode (NM1) predicted by the ANM can effectively reproduce the functionally-relevant conformational change described by PC1 [68]. Indeed, structural variations described by the first PCs are well covered by the low-frequency modes, with a large cumulative overlap of 0.81 between NM1-NM3 and PC1 and a RMSIP of 0.66 between subspaces defined by NM1-NM3 and PC1-PC3 (for NM1-NM10 and PC1-PC10 the RMSIP is 0.58).

Comparison between NMs and EDs was also made by calculating the overlap between corresponding modes. As it can be seen in Fig 2C, there is significant overlap between NM modes and EDs from Sim1, with a particularly large overlap of 0.82 between NM1 and ED3 (0.69 between ED1 and NM1 from Sim2). Remarkably, a high RMSIP of 0.72 is also obtained between subspaces defined by NM1-NM3 and ED1-ED3 for both Sim1 and Sim2 (for NM1-NM10 and ED1-ED10 the RMSIP is 0.76 for Sim1 and 0.74 for Sim2). Therefore, it can be said that the employed coarse-grained ANM can reproduce fairly well the conformational space explored by thermolysin during the two independent MD simulations.

In conclusion, the results show that both MD simulations and ANM provide a reasonable description of thermolysin internal dynamics, particularly the subspace of collective motions with functional relevance. This prompted the use of ENM-based methods to study other evolutionarily-related MPs and to quantitatively compare their internal dynamics. The obtained results will be discussed in following section.

### Structural and Dynamical Alignments

Structure- and dynamics-based alignments of a representative set of 13 MA clan proteins (Table 2) was made using the DaliLite and ALADYN web-servers, respectively, as described in Methods section. The employed algorithm in ALADYN is based on the β-Gaussian ENM, where a two-nodes per residue representation is used. Using this approach, the inclusion of additional metal ion nodes could not be evaluated as in the previous section for thermolysin ANM. However, given the structural heterogeneity of clan representatives, the difference between their respective dynamical profiles is expected to surpass the local changes produced in each protein by the inclusion of metal nodes.

**Table 2:** Set of 13 representative metalloproteases of the MA Clan.

MEROPS Sub-clan	MEROPS Family	Protein	PDB ID	CATH Superfamily	SCOP Family
MA(E)	M4	Thermolysin	1L3F	1.10.390.10; 3.10.170.10	55490
MA(E)	M1	Leukotriene A4 hydrolase	1H19	1.10.390.10	64338
MA(E)	M3	Neurolysin	1I1I	1.10.1370.10; 3.40.390.10; 1.20.1050.40	55505
MA(E)	M32	Carboxypeptidase *Pfu*	1KA4	n.a.	55505
MA(E)	M2	Angiotensin Converting Enzyme	1O8A	n.a.	55505
MA(E)	M27	Botulinum neurotoxin type A	3BON	n.a.	55512
MA(M)	M7	Extracellular small neutral protease	1C7K	3.40.390.10	55487
MA(M)	M35	Peptidyl-Lys metalloendopeptidase	1G12	3.40.390.10	55487
MA(M)	M8	Leishmanolysin	1LML	3.90.132.10; 3.10.170.20; 2.30.34.10; 2.10.55.10	55499
MA(M)	M10(A)	Interstitial collagenase	1CGE	3.40.390.10	55528
MA(M)	M10(B)	Serralysin	1AKL	n.a.; 2.150.10.10	55508
MA(M)	M12(A)	Astacin	1AST	3.40.390.10	55516
MA(M)	M12(B)	Snake venom metalloproteinase adamalysin-2	1IAG	3.40.390.10	55519

**Fig 3:**
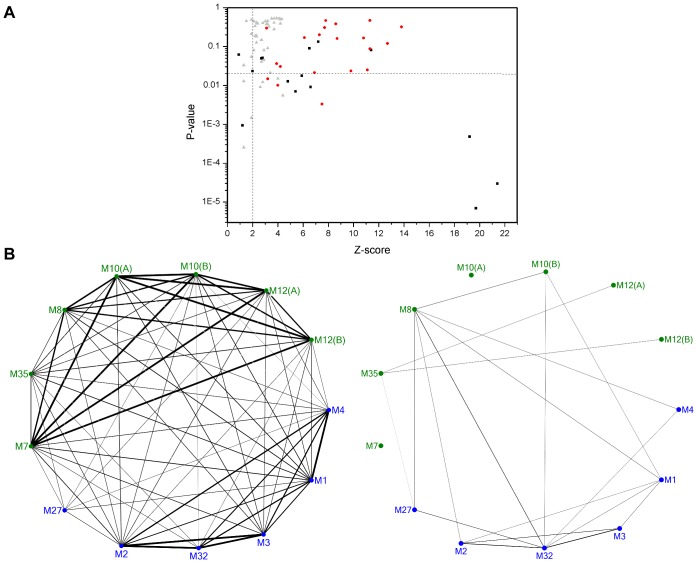
Dynamical and structural variability of MA clan representative structures. (A) Mapping of structural (Z-score) and dynamical (P-values) alignment scores obtained for the pairs of MP structures. Red circles: Metzincin pairs; Black squares: Gluzincin pairs; Gray triangles: Mixed pairs. Dashed lines indicate threshold values for structural (vertical) and dynamical (horizontal) similarity. Labeled pairs were selected for further inspection. (B) Graph representation of the structural (left) and dynamical (right) similarity between MP representatives (Blue: Gluzincins; Green: Metzincins). Edge width is proportional to the corresponding Z-score and P-value and only edges with width values above the corresponding thresholds are represented.

The scores obtained for each of the resulting 78 aligned pairs are shown in Fig 3 and Table B in S1 File, being classified into three distinct groups: 1) MA(E), with both proteins belonging to the MA(E) subclan; 2) MA(M), for proteins belonging to the MA(M) subclan and; 3) Mixed, with each protein belonging to different subclans. Given that each representative protein is not a homologue of the remaining representative proteins [2], the simplified mapping of scores shown in Fig 3A can be seen as an additional layer to the typical representation of protein relatedness in terms of sequence similarity, providing a characterization of the clan in terms of structural and dynamical diversity. As it will be discussed below, it allows for the identification of unnoticed functional similarities between distinct proteins and provides a description of how structure and internal dynamics of proteins are related within a given clan. It can be useful, *e*.*g*. in the field of structural genomics, where protein function assignment could be made based not only on sequence and structural similarity, but also on information obtained from dynamics-based alignments with a set of proteins with known catalytic function.

The threshold values to consider a pair either structurally or dynamically similar are based on the employed methods. Overall, the majority of pairs analyzed are structurally similar; with 87% of pairs having Z-scores > 2.0 [72]. However, 69% of pairs are not dynamically similar, since they have P-values > 0.02 [51]. Regarding the structural conservation of the three types of pairs considered, all 21 MA(M) pairs are structurally similar while for MA(E) pairs this is the case for 87% of the 15 pairs. In the case of mixed pairs, 81% of the 42 are structurally similar, although with Z-scores clustered near the threshold of structural similarity. Regarding the internal dynamics of subclan members, remarkably, only 14% of MA(M) pairs have dynamical similarity, while 53% of MA(E) pairs also present it. In the case of mixed pairs, only 17% have dynamical similarity. Since the internal dynamics of proteins are ultimately dependent on their structure, structural similarity is expected to be related with dynamical similarity, *i*.*e*. pairs with higher structural similarity scores would tend to have higher dynamical similarity scores. However, no correlation is apparent between Z-scores and P-values for all analyzed pairs, although it is noted that pairs with Z-scores > 19.0 are associated with a high dynamical similarity scores (P-value < 0.001), corresponding to MA(E) pairs M3-M2, M3-M32 and M32-M2. As it will be discussed below, MA(E) pairs M32-M8 and M32-M27 are particularly relevant since their respective alignments have Z-Scores < 2.0 and P-values < 0.001. In the case of MA(M) pairs, there is an apparent inverted relation between dynamical and structural similarities, since pairs with lower structural similarity (lower Z-scores) show higher dynamical similarity (lower P-values), *e*.*g*. pair members M35-M12(B), M35-M12(A), M8-M10(A).

A similar analysis was made by Carnevale *et al*., where no general trend between structural and dynamical similarity scores for pairs of proteases from different clans and catalytic chemistries was found [6]. In that case, no specific correlation was expected since the analyzed representatives were considered to have different evolutionary origin and therefore share minimal structural homology. Nonetheless, structural and dynamical similarity was identified for some pairs, with the authors arguing for convergent evolutionary pressure to be at play in such cases. It was therefore suggested that in cases where structurally variability is observed, a compensatory mechanism for dynamical conservation could maintain the catalytic capacity of the proteins. Conversely, since in this study MP representatives are from the same clan and therefore have assumed common ancestry, no structural or dynamical similarity between two representatives is expected to reflect a divergent evolutionary process. Given that structural and dynamical similarities are apparently not correlated in this case, it is suggested that selective pressure is acting independently on structure and internal dynamics.

Fig 3B provides an overview of how individual representatives are related in terms of structural and dynamical similarity. Only pairs considered to be structurally- or dynamically- similar are linked in the weighed graphs, where edge width is related to Z-score and P-value. In terms of structural similarity, members are more connected to representatives belonging to the same subclan, but overall there are also inter-subclan connections, reflecting the structural conservation across the entire clan. There is one exception for representative M27, *Botulinum* Neurotoxin Type A Light Chain, which shows low connectivity to the remaining representatives.

Regarding dynamical similarity, there is less connectivity between representatives, which may reflect divergence of internal dynamics along the clan. Also, subclan similarity is less pronounced as it can be seen for the case of, *e*.*g*. representative M8, which has higher connectivity with MA(E) subclan members than with those of MA(M) subclan where it belongs to. Notable exceptions are representatives Extracellular Small Neutral Protease (M7) and Interstitial Collagenase (M10(A)) that share no dynamical similarity with the remaining MP representatives. These two proteins are relatively small in size; Extracellular Small Neutral Protease is a 132-residue long protein and the structure of Interstitial Collagenase corresponds only to the 168-residue long catalytic domain. Although their internal dynamics are also characterized by hinge-bending motions (not shown), the amplitude of motions of their smaller domains has no correspondence with the motions of the larger domains of the remaining representatives.

These results provide a quantitative measure of the structural and dynamical similarity that characterizes MA clan members and provides a “horizontal” view on MP evolution. In order to understand if there is a functional basis for the conservation or divergence of such features, a chosen set of alignments was further inspected. For pairs with high structural and dynamical similarity M32-M2, M3-M32 and M3-M2, the structural homology between the corresponding representatives Angiotensin Converting Enzyme (M2), Neurolysin (M3) and Carboxypeptidase *Pfu* (M32) had been previously reported [107–109]. Indeed, these proteins are grouped in the same SCOP family and their high dynamical similarity can be related directly to their structural resemblance. Nonetheless, Angiotensin Converting Enzyme and Carboxypeptidase *Pfu* have no attributed CATH codes and Neurolysin presents three domains, including the common 3.40.390.10 domain found in other representatives. The alignments of Neurolysin and Carboxypeptidase *Pfu*, which represent the pair with highest dynamical similarity, were chosen for further inspection. Neurolysin is a 681 residue-long endopeptidase that cleaves the 13-residue peptide neurotensin but it has also activity towards a diverse set of oligopeptide sequences [107]. Carboxypeptidase *Pfu* is a 499-residue long thermostable carboxypeptidase homodimer with broad substrate specificity [108].

**Fig 4:**
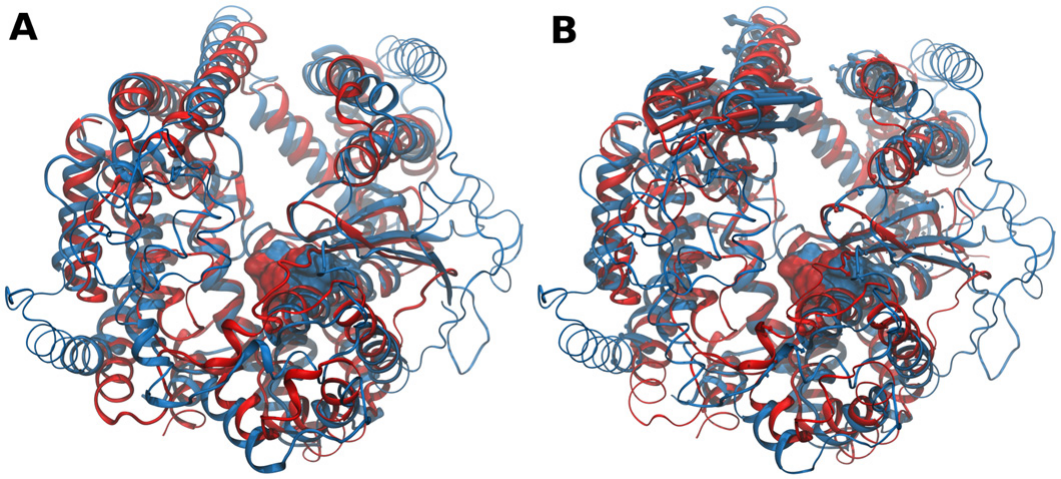
Structure- and dynamics-based alignments obtained for pair M3-M32. (A) Structure-based and (B) Dynamics-based alignment of M3 representative Neurolysin (blue, PDB ID: 1I1I) and M32 representative Carboxypeptidase *Pfu* (red, PDB ID: 1KA4). Produced alignments were obtained using the DaliLite and ALADYN web-servers (see Methods). Aligned residues colored in cartoon representation, non-aligned residues in colored ribbons and active site residues in surface representations (Neurolysin: H474, E475, H478 and E503; Carboxypeptidase *Pfu* H269, E270, H273 and E299). Colored arrows indicate modes of motion of aligned portions along the first mode.

Both structures are mainly α-helical in content and characterized by a deep narrow channel that divide the structures into two domains, with a wider opening at one end and with the active site located at the bottom. This prevents activity towards large, folded substrates. Their structure-based alignment is shown in Fig 4A. The alignment produces a RMSD of 3.7 Å for 449 amino acids used with 15% sequence identity. Aligned regions consist on core regions surrounding the active sites and α-helices that constitute the channel base and walls. The respective dynamics-based alignment, shown in Fig 4B, produces a lower number of equivalent residues, with RMSD of 2.8 Å for 371 amino acids with 13.5% sequence identity (RMSIP of 0.870). It reveals that these regions undergo very similar deformations resembling hinge motions, most likely corresponding to channel opening for substrate access. The aligned portions in both structure- and dynamics-based alignments have high degree of identity.

The motions produced by the employed β-Gaussian ENM are in agreement with findings reported for each MP. Indeed, hinge movements of Carboxypeptidase *Pfu* sub-domains were previously argued to be involved in channel closure and this conformational change was proposed to have a functional role, namely to extend the number of interactions with the substrate [108]. In the case of Neurolysin, it was suggested that both domains are rigid but present some mobility in relation to each other due to looser packing at the base of the channel [107]. In both cases, the amplitude of hinge movements is restricted due to the presence of cross-domain segments that tighten the channel at one end and limit the access of longer substrates to the buried active site. Remarkably, Carboxypeptidase *Pfu* cross-domain α-helix (α_4_, residues 81–99), that contains the conserved R92 considered to have a crucial role in substrate-length restriction, is almost perfectly aligned with an equivalent cross-domain α-helix of Neurolysin (α_5_, residues 137–152) in both structure- and dynamics-based alignments. Moreover, it is noted that Neurolysin residue Lys148 and the conserved Arg92 of Carboxypeptidase *Pfu* are very close positioned in the dynamics-based alignment of the respective structures, that together with sharing the chemical nature suggests their functional equivalence (Fig B in S2 File). Therefore, the conservation of internal dynamics between these two MPs may not be only a direct consequence of their structural similarity, but may also have a functional basis. Specifically, the requirement to have specific channel opening amplitudes in order to restrain substrate-length, while more local variations in structure allow for distinct specificities in terms of substrate sequence recognition and binding.

Further support for the conservation of internal dynamics between these two MPs comes from the findings reported for Angiotensin Converting Enzyme, which shares high structural and dynamical similarity with both Neurolysin and Carboxypeptidase *Pfu* [110,111]. The authors showed that for this MP hinge-bending motions have a functional role, since channel opening allows for substrate access to the active site. Furthermore, it was shown that these motions are conserved between other M2 family members. Finally, the presumed dynamical resemblance between M2 family members and another member of the M3 family, Carboxypeptidase *Dcp*, has been previously noted [112]. Together with these findings, results obtained for M2, M3 and M32 representatives suggest that conservation of internal dynamics is not limited to homologues, but that it can be extended for other families that share some structural similarity.

**Fig 5:**
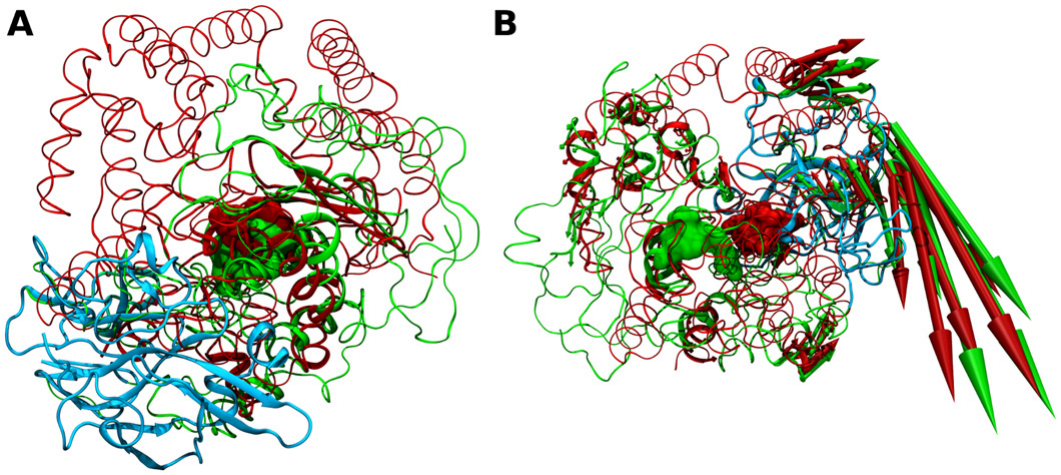
Structure- and dynamics-based alignment obtained for pair M32-M8. (A) Structure-based and (B) Dynamics-based alignment of M8 representative Leishmanolysin (green, PDB ID: 1LML) and M32 representative Carboxypeptidase *Pfu* (red, PDB ID: 1KA4). Produced alignments were obtained using the DaliLite and ALADYN web-servers (see Methods). Aligned residues colored in cartoon representation, non-aligned residues in colored ribbons and active site residues in surface representations (Leishmanolysin: H264, E265, H268, H334 and; Carboxypeptidase Pfu H269, E270, H273 and E299). Colored arrows indicate modes of motion of aligned portions along the first mode. Leishmanolysin C-terminal domain in cyan colored tube representation.

Pairs M32-M8, M27-M8 and M32-M27 were also analyzed due to their high dynamical similarity with no structural similarity. Both Carboxypeptidase *Pfu* (M32) and *Botulinum* Neurotoxin Type A (M27) belong to the Gluzincin subclan but have no attributed CATH numbering. Leishmanolysin (M8) belongs to the Metzincin subclan and has four CATH domains, with the particular feature of lacking the conserved 3.40.390.10 domain shared by other Metzincin subclan representatives. The pair of Leishmanolysin and Carboxypeptidase *Pfu* was chosen for further inspection, as it corresponds to the only Mixed pair with very high dynamical similarity. Leishmanolysin (also termed GP63) is a 478 residue-long surface glycoprotein from *Leishmania major* that occurs as a dimer and has activity towards a wide variety of peptidic substrates [113]. It adopts a predominantly compact fold composed of mostly β-sheet secondary structure elements, in contrast with the predominant α-helical structure adopted by Carboxypeptidase *Pfu* [114]. The structure differs from Carobxypeptidase *Pfu* and other MPs as it is composed of three domains: the N-terminal domain that contains the active site HEXXH sequence motif; the central domain, that presents an unique 62 residue-long insertion between the conserved Glycine of the HEXXHXXGXXH/D metzincin sequence motif and the third active site residue Histidine; and the C-terminal domain, which contains the membrane anchoring point and is composed mainly of β-strand and random coil elements, being positioned at one end of the active site cleft base. Both N- and central domain form the active site cleft. EDA of Leishmanolysin MD trajectories revealed that large-scale, collective motions dominate the conformational changes explored by the protein. These consist in hinge-bending motions characterized by rigid body movements of the N-terminal domain relative to the central and C-terminal domains, with the hinge axis located at base of the active site cleft [115]. The corresponding structure-based alignment is shown in Fig 5A, with a RMSD of 4.6 Å for 88 amino acids used with 9% sequence identity. Unlike the pairs with high structural and dynamical similarity with aligned regions spanning almost the entirety of the structures, the aligned portions of Leishmanolysin and Carboxypeptidase *Pfu* are restricted to the N-terminal domain, including the α-helix containing the active site HEXXH sequence motif. Indeed, this was typically observed for other non-structurally similar pairs of MPs [1]. The overall orientation of the structures is kept, with the substrate-binding pockets and active residues being almost identically positioned in both structures and with the positioning of the C-terminal domain of Leishmanolysin at the more open end of Carboxypeptidase *Pfu* channel. Dynamics-based alignment shown in Fig 5B produces a slightly higher number of equivalent residues, with an RMSD of 3.3 Å for 91 amino acids with 7.7% sequence identity (RMSIP of 0.832). Remarkably, the dynamics-based alignment results in a complete horizontal rotation of Leishmanolysin in relation to Carboxypeptidase *Pfu* when compared to the structure-based alignment. This results in the positioning of its C-terminal domain at the cross-domain segments that constitute the more closed end of Carboxypeptidase *Pfu* channel and the active sites become approximately 10 Å apart from each other. Nonetheless, the substrate binding-pocket of both MPs retain their orientation and regions undergoing hinge-bending motions are almost identically positioned, indicating their dynamical equivalence. This is also observed in the structure-based and dynamics-based alignments produced for Leishmanolysin and *Botulinum* Neurotoxin Type A (data not shown), thus suggesting that there is conservation of internal dynamics even when remarkable structural differences between members occur. Moreover, this conservation has a functional basis, namely to allow for proper orientation of interactions between the proteins and their substrates. Therefore, the relatedness of Leishmanolysin, *Botulinum* Neurotoxin Type A and Carboxypeptidase *Pfu*, which has so far been only considered at the clan level, becomes evident when their internal dynamics are taken into account.

## Conclusions

This work provides a quantitative characterization of the structural and dynamical diversity occurring within the MEROPS MA clan of MPs. It shows that metalloproteases of this clan have distinct dynamical profiles despite their overall structural similarity. Also, it is shown that in cases where high dynamical similarity is observed, the predominant modes correspond to hinge-bending motions associated with substrate-binding. These motions are functionally relevant and appear to be conserved in the clan even when remarkable structural differences between its members occur. Therefore, besides providing a description of the structural and dynamical features of a set of proteins, this type of analysis can also provide new insights on enzyme function that remained unnoticed so far. For metalloproteases, it is suggested that the need to maintain proper substrate interactions has an important role on the conservation of their internal dynamics. Therefore, the type of interactions between a protein and its ligand and the associated motions should be more carefully considered when comparing the internal dynamics of a diverse set of functionally distinct proteins. Together, this work contributes to the development of simple and effective approaches that incorporate quantitative analysis of dynamical similarity between proteins to study the evolution of metalloprotease internal dynamics and the factors governing them.

## Supporting Information

S1 File Thermolysin structures (Uniprot ID: P00800) retrieved from the PDB. Unbound structures in bold. Reference structure for PC, NM and MD simulations in bold underlined (Table A). List of Z-scores and P-values obtained for the alignments of MP representative structures (Table B). (DOCX)

S2 File Root Mean Square Deviations of residue C^α^ obtained for Sim1 and Sim2 (Fig A). Dynamics-based alignment of Neurolysin (Blue, PDB ID: 1I1I) and Carboxypeptidase Pfu (red, PDB ID: 1KA4). Neurolysin K148 and Carboxypeptidase Pfu R92 in bond representation. Active site residues in colored surface representations (Neurolysin: H474, E 475, H478 and E503; Carboxypeptidase Pfu H269, E270, H273 and E299) **(Fig B)**.
(DOCX)

## References

[1] Barrett AJ. Handbook of Proteolytic Enzymes 3rd ed. Handbook of Proteolytic Enzymes. Elsevier; 2013 pp. 325–370. 10.1016/B978-0-12-382219-2.00077-6

[2] Rawlings ND, Waller M, Barrett AJ, Bateman A. MEROPS: the database of proteolytic enzymes, their substrates and inhibitors. Nucleic Acids Res. 2014;42: D503–9. 10.1093/nar/gkt953 24157837 PMC3964991

[3] Murzin A, Brenner S. SCOP: a structural classification of proteins database for the investigation of sequences and structures. J Mol Biol. 1995;247: 536–540. 10.1016/S0022-2836(05)80134-2 7723011

[4] Sillitoe I, Cuff AL, Dessailly BH, Dawson NL, Furnham N, Lee D, et al New functional families (FunFams) in CATH to improve the mapping of conserved functional sites to 3D structures. Nucleic Acids Res. 2013;41: D490–8. 10.1093/nar/gks1211 23203873 PMC3531114

[5] Tyndall J, Nall T, Fairlie D. Proteases universally recognize beta strands in their active sites. Chem Rev. 2005;105: 973–1000. 10.1021/cr040669e 15755082

[6] Carnevale V, Raugei S, Micheletti C, Carloni P. Convergent Dynamics in the Protease Enzymatic Superfamily. 2006; 9766–9772. 10.1021/ja060896t 16866533

[7] Gagné D, Doucet N. Structural and functional importance of local and global conformational fluctuations in the RNase A superfamily. FEBS J. 2013;280: 5596–607. 10.1111/febs.12371 23763751

[8] García-Meseguer R, Martí S, Ruiz-Pernía JJ, Moliner V, Tuñón I. Studying the role of protein dynamics in an SN2 enzyme reaction using free-energy surfaces and solvent coordinates. Nat Chem. Nature Publishing Group; 2013;5: 566–71. 10.1038/nchem.1660 23787745

[9] Hammes-Schiffer S, Benkovic SJ. Relating protein motion to catalysis. Annu Rev Biochem. 2006;75: 519–41. 10.1146/annurev.biochem.75.103004.142800 16756501

[10] McGowan LC, Hamelberg D. Conformational plasticity of an enzyme during catalysis: intricate coupling between cyclophilin A dynamics and substrate turnover. Biophys J. Biophysical Society; 2013;104: 216–26. 10.1016/j.bpj.2012.11.3815 PMC3540238 23332074

[11] Luk LYP, Javier Ruiz-Pernía J, Dawson WM, Roca M, Loveridge EJ, Glowacki DR, et al Unraveling the role of protein dynamics in dihydrofolate reductase catalysis. Proc Natl Acad Sci U S A. 2013;110: 16344–9. 10.1073/pnas.1312437110 24065822 PMC3799346

[12] Glowacki D, Harvey J, Mulholland A. Taking Ockham’s razor to enzyme dynamics and catalysis. Nat Chem. 2012;4: 169–176. 10.1038/NCHEM.1244 22354430

[13] Hammes-Schiffer S. Catalytic efficiency of enzymes: a theoretical analysis. Biochemistry. 2013;52: 2012–20. 10.1021/bi301515j 23240765 PMC3619019

[14] Henzler-Wildman K a, Lei M, Thai V, Kerns SJ, Karplus M, Kern D. A hierarchy of timescales in protein dynamics is linked to enzyme catalysis. Nature. 2007;450: 913–6. 10.1038/nature06407 18026087

[15] Jones AR, Levy C, Hay S, Scrutton NS. Relating localized protein motions to the reaction coordinate in coenzyme BII-dependent enzymes. FEBS J. 2013;280: 2997–3008. 10.1111/febs.12223 23462350

[16] Hay S, Scrutton NS. Good vibrations in enzyme-catalysed reactions. Nat Chem. Nature Publishing Group; 2012;4: 161–8. 10.1038/nchem.1223 22354429

[17] Schwartz SD, Schramm VL. Enzymatic transition states and dynamic motion in barrier crossing. Nat Chem Biol. 2009;5: 551–8. 10.1038/nchembio.202 19620996 PMC2859820

[18] Ma B, Nussinov R. Enzyme dynamics point to stepwise conformational selection in catalysis. Curr Opin Chem Biol. Elsevier Ltd; 2010;14: 652–9. 10.1016/j.cbpa.2010.08.012 PMC6407632 20822947

[19] Liu Y, Bahar I. Sequence evolution correlates with structural dynamics. Mol Biol Evol. 2012;29: 2253–63. 10.1093/molbev/mss097 22427707 PMC3424413

[20] Marsh J a, Teichmann S a. Parallel dynamics and evolution: Protein conformational fluctuations and assembly reflect evolutionary changes in sequence and structure. Bioessays. 2014;36: 209–18. 10.1002/bies.201300134 24272815

[21] Maguid S, Fernandez-Alberti S, Ferrelli L, Echave J. Exploring the common dynamics of homologous proteins. Application to the globin family. Biophys J. Elsevier; 2005;89: 3–13. 10.1529/biophysj.104.053041 PMC1366528 15749782

[22] Maguid S, Fernández-Alberti S, Parisi G, Echave J. Evolutionary conservation of protein backbone flexibility. J Mol Evol. 2006;63: 448–57. 10.1007/s00239-005-0209-x 17021932

[23] Raimondi F, Orozco M, Fanelli F. Deciphering the deformation modes associated with function retention and specialization in members of the Ras superfamily. Structure. Elsevier Ltd; 2010;18: 402–14. 10.1016/j.str.2009.12.015 20223222

[24] Marcos E, Crehuet R, Bahar I. On the conservation of the slow conformational dynamics within the amino acid kinase family: NAGK the paradigm. PLoS Comput Biol. 2010;6: e1000738 10.1371/journal.pcbi.1000738 20386738 PMC2851564

[25] Luebbering EK, Mick J, Singh RK, Tanner JJ, Mehra-Chaudhary R, Beamer LJ. Conservation of functionally important global motions in an enzyme superfamily across varying quaternary structures. J Mol Biol. Elsevier Ltd; 2012;423: 831–46. 10.1016/j.jmb.2012.08.013 22935436

[26] Maguid S, Fernandez-Alberti S, Echave J. Evolutionary conservation of protein vibrational dynamics. Gene. 2008;422: 7–13. 10.1016/j.gene.2008.06.002 18577430

[27] Zen A, Carnevale V, Lesk A, Micheletti C. Correspondences between low‐energy modes in enzymes: Dynamics‐based alignment of enzymatic functional families. Protein Sci. 2008;17: 918–929. 10.1110/ps.073390208 18369194 PMC2327282

[28] Pang A, Arinaminpathy Y, Sansom MSP, Biggin PC. Comparative molecular dynamics—similar folds and similar motions? Proteins. 2005;61: 809–22. 10.1002/prot.20672 16231327

[29] Leo-Macias A, Lopez-Romero P, Lupyan D, Zerbino D, Ortiz AR. An analysis of core deformations in protein superfamilies. Biophys J. 2005;88: 1291–9. 10.1529/biophysj.104.052449 15542556 PMC1305131

[30] Velázquez-Muriel J a, Rueda M, Cuesta I, Pascual-Montano A, Orozco M, Carazo J-M. Comparison of molecular dynamics and superfamily spaces of protein domain deformation. BMC Struct Biol. 2009;9: 6 10.1186/1472-6807-9-6 19220918 PMC2666742

[31] Tobi D, Bahar I. Structural changes involved in protein binding correlate with intrinsic motions of proteins in the unbound state. Proc Natl Acad Sci U S A. 2005;102: 18908–18913. 16354836 10.1073/pnas.0507603102 PMC1323175

[32] Bahar I, Lezon TR, Yang L-W, Eyal E. Global dynamics of proteins: bridging between structure and function. Annu Rev Biophys. 2010;39: 23–42. 10.1146/annurev.biophys.093008.131258 20192781 PMC2938190

[33] Hollup SM, Fuglebakk E, Taylor WR, Reuter N. Exploring the factors determining the dynamics of different protein folds. Protein Sci. 2011;20: 197–209. 10.1002/pro.558 21086444 PMC3047076

[34] Echave J. Why are the low-energy protein normal modes evolutionarily conserved? Pure Appl Chem. 2012;84: 1931–1937. 10.1351/PAC-CON-12-02-15

[35] Liberles D a, Teichmann S a, Bahar I, Bastolla U, Bloom J, Bornberg-Bauer E, et al The interface of protein structure, protein biophysics, and molecular evolution. Protein Sci. 2012;21: 769–85. 10.1002/pro.2071 22528593 PMC3403413

[36] Echave J, Fernández FM. A perturbative view of protein structural variation. Proteins. 2010;78: 173–80. 10.1002/prot.22553 19731380

[37] Lai J, Jin J, Kubelka J, Liberles D a. A phylogenetic analysis of normal modes evolution in enzymes and its relationship to enzyme function. J Mol Biol. Elsevier Ltd; 2012;422: 442–59. 10.1016/j.jmb.2012.05.028 PMC3423504 22651983

[38] Ramanathan A, Agarwal PK. Evolutionarily conserved linkage between enzyme fold, flexibility, and catalysis. PLoS Biol. 2011;9: e1001193 10.1371/journal.pbio.1001193 22087074 PMC3210774

[39] Keskin O, Jernigan R, Bahar I. Proteins with similar architecture exhibit similar large-scale dynamic behavior. Biophys J. 2000;78: 2093–2106. 10733987 10.1016/S0006-3495(00)76756-7 PMC1300801

[40] Münz M, Lyngsø R, Hein J, Biggin PC. Dynamics based alignment of proteins: an alternative approach to quantify dynamic similarity. BMC Bioinformatics. 2010;11: 188 10.1186/1471-2105-11-188 20398246 PMC2868010

[41] Bhabha G, Ekiert DC, Jennewein M, Zmasek CM, Tuttle LM, Kroon G, et al Divergent evolution of protein conformational dynamics in dihydrofolate reductase. Nat Struct Mol Biol. Nature Publishing Group; 2013;20: 1243–9. 10.1038/nsmb.2676 PMC3823643 24077226

[42] Dellus-Gur E, Toth-Petroczy A, Elias M, Tawfik DS. What makes a protein fold amenable to functional innovation? Fold polarity and stability trade-offs. J Mol Biol. Elsevier Ltd; 2013;425: 2609–21. 10.1016/j.jmb.2013.03.033 23542341

[43] Gatti-Lafranconi P, Hollfelder F. Flexibility and reactivity in promiscuous enzymes. Chembiochem. 2013;14: 285–92. 10.1002/cbic.201200628 23362046

[44] Münz M, Hein J, Biggin PC. The role of flexibility and conformational selection in the binding promiscuity of PDZ domains. PLoS Comput Biol. 2012;8: e1002749 10.1371/journal.pcbi.1002749 23133356 PMC3486844

[45] Tokuriki N, Tawfik D. Protein dynamism and evolvability. Science. 2009;324: 203–207. 10.1126/science.1169375 19359577

[46] Pandini A, Mauri G, Bordogna A, Bonati L. Detecting similarities among distant homologous proteins by comparison of domain flexibilities. Protein Eng Des Sel. 2007;20: 285–99. 10.1093/protein/gzm021 17573407

[47] Gherardini PF, Helmer-Citterich M. Structure-based function prediction: approaches and applications. Brief Funct Genomic Proteomic. 2008;7: 291–302. 10.1093/bfgp/eln030 18599513

[48] Hensen U, Meyer T, Haas J, Rex R, Vriend G, Grubmüller H. Exploring protein dynamics space: the dynasome as the missing link between protein structure and function. PLoS One. 2012;7: e33931 10.1371/journal.pone.0033931 22606222 PMC3350514

[49] Tobi D. Dynamics alignment: comparison of protein dynamics in the SCOP database. Proteins. 2012;80: 1167–76. 10.1002/prot.24017 22275069

[50] Micheletti C. Comparing proteins by their internal dynamics: exploring structure-function relationships beyond static structural alignments. Phys Life Rev. Elsevier B.V.; 2013;10: 1–26. 10.1016/j.plrev.2012.10.009 23199577

[51] Potestio R, Aleksiev T, Pontiggia F, Cozzini S, Micheletti C. ALADYN: a web server for aligning proteins by matching their large-scale motion. Nucleic Acids Res. 2010;38: W41–5. 10.1093/nar/gkq293 20444876 PMC2896196

[52] Tobi D. Normal Mode Dynamics Comparison of Proteins. 2014;40700: 1118–1125. 10.1002/ijch.201300142

[53] Bakan A, Meireles LM, Bahar I. ProDy: protein dynamics inferred from theory and experiments. Bioinformatics. 2011;27: 1575–7. 10.1093/bioinformatics/btr168 21471012 PMC3102222

[54] Bakan A, Dutta A, Mao W, Liu Y, Chennubhotla C, Lezon TR, et al Evol and ProDy for bridging protein sequence evolution and structural dynamics. Bioinformatics. 2014;30: 2681–3. 10.1093/bioinformatics/btu336 24849577 PMC4155247

[55] Berman HM, Westbrook J, Feng Z, Gilliland G, Bhat TN, Weissig H, et al The Protein Data Bank. Nucleic Acids Res. 2000;28: 235–42. 10592235 10.1093/nar/28.1.235 PMC102472

[56] Hess B, Kutzner C, Van Der Spoel D, Lindahl E. GROMACS 4: Algorithms for highly efficient, load-balanced, and scalable molecular simulation. J Chem Theory Comput. 2008;4: 435–447. 26620784 10.1021/ct700301q

[57] Berendsen HJC, van der Spoel D, van Drunen R. GROMACS: A message-passing parallel molecular dynamics implementation. Comput Phys Commun. 1995;91: 43–56. 10.1016/0010-4655(95)00042-E

[58] Lindahl E, Hess B, Van Der Spoel D. GROMACS 3 . 0 : a package for molecular simulation and trajectory analysis. J Mol Model. 2001;43: 306–317.

[59] Lindorff-Larsen K, Piana S, Palmo K, Maragakis P, Klepeis JL, Dror RO, et al Improved side-chain torsion potentials for the Amber ff99SB protein force field. Proteins Struct Funct Bioinforma. 2010;78: 1950–1958. 10.1002/prot.22711 PMC2970904 20408171

[60] Berendsen HJC, Grigera JR, Straatsma TP. The Missing Term in Effective Pair Potentials. J Phys Chem. 1987;91: 6269–6271. 10.1021/j100308a038

[61] Bussi G, Donadio D, Parrinello M. Canonical sampling through velocity rescaling. J Chem Phys. 2007;126: 014101 10.1063/1.2408420 17212484

[62] Berendsen HJC, Postma JPM, van Gunsteren WF, DiNola A, Haak JR. Molecular dynamics with coupling to an external bath. J Chem Phys. 1984;81: 3684.

[63] Hess B, Bekker H, Berendsen HJC, Fraaije JGEM. LINCS: A linear constraint solver for molecular simulations. J Comput Chem. 1997;18: 1463–1472. 10.1002/(SICI)1096-987X(199709)18:12<1463::AID-JCC4>3.0.CO;2-H

[64] Xu D, Cui Q, Guo H. Quantum mechanical/molecular mechanical studies of zinc hydrolases. Int Rev Phys Chem. 2014;33: 1–41. 10.1080/0144235X.2014.889378

[65] Holland DR, Hausrath AC, Juers D, Matthews BW. Structural analysis of zinc substitutions in the active site of thermolysin. Protein Sci. 1995;4: 1955–1965. 10.1002/pro.5560041001 8535232 PMC2142975

[66] Doruker P, Atilgan AR, Bahar I. Dynamics of proteins predicted by molecular simulations and analytical approaches: Application to alpha-amylase inhibitor. Proteins Struct Funct Genet. 2000;40: 512–524. 10.1002/1097-0134(20000815)40:3<512::AID-PROT180>3.0.CO;2-M 10861943

[67] Atilgan a R, Durell SR, Jernigan RL, Demirel MC, Keskin O, Bahar I. Anisotropy of fluctuation dynamics of proteins with an elastic network model. Biophys J. 2001;80: 505–515. 11159421 10.1016/S0006-3495(01)76033-X PMC1301252

[68] Tama F, Sanejouand YH. Conformational change of proteins arising from normal mode calculations. Protein Eng. 2001;14: 1–6. 11287673 10.1093/protein/14.1.1

[69] schweiler R. Collective protein dynamics and nuclear spin relaxation. J Chem Phys. 1995;102: 3396.

[70] Amadei A, Ceruso MA, Di Nola A. On the convergence of the conformational coordinates basis set obtained by the Essential Dynamics analysis of proteins’ molecular dynamics simulations. Proteins Struct Funct Genet. 1999;36: 419–424. 10.1002/(SICI)1097-0134(19990901)36:4<419::AID-PROT5>3.0.CO;2-U 10450083

[71] Hess B. Convergence of sampling in protein simulations. Phys Rev E—Stat Nonlinear, Soft Matter Phys. 2002;65: 1–10. 10.1103/PhysRevE.65.031910 11909112

[72] Holm L, Park J. DaliLite workbench for protein structure comparison. Bioinformatics. 2000;16: 566–567. 10980157 10.1093/bioinformatics/16.6.566

[73] Micheletti C, Carloni P, Maritan A. Accurate and efficient description of protein vibrational dynamics: comparing molecular dynamics and Gaussian models. Proteins. 2004;55: 635–45. 10.1002/prot.20049 15103627

[74] Zen A, Carnevale V, Lesk AM, Micheletti C. Correspondences between low-energy modes in enzymes: dynamics-based alignment of enzymatic functional families. Protein Sci. 2008;17: 918–929. 10.1110/ps.073390208 18369194 PMC2327282

[75] Fuglebakk E, Reuter N, Hinsen K. Evaluation of protein elastic network models based on an analysis of collective motions. J Chem Theory Comput. 2013;9: 5618–5628. 10.1021/ct400399x 26592296

[76] Pelmenschikov V, Blomberg MR a, Siegbahn PEM. A theoretical study of the mechanism for peptide hydrolysis by thermolysin. J Biol Inorg Chem. 2002;7: 284–98. 10.1007/s007750100295 11935352

[77] Blumberger J, Lamoureux G, Klein ML. Peptide Hydrolysis in Thermolysin: Ab Initio QM/MM Investigation of the Glu143-Assisted Water Addition Mechanism. J Chem Theory Comput. 2007;3: 1837–1850. 26627626 10.1021/ct7000792

[78] Bakan A, Bahar I. The intrinsic dynamics of enzymes plays a dominant role in determining the structural changes induced upon inhibitor binding. Proc Natl Acad Sci U S A. 2009;106: 14349–54. 10.1073/pnas.0904214106 19706521 PMC2728110

[79] Yang L, Song G, Carriquiry A, Jernigan RL. Close correspondence between the motions from principal component analysis of multiple HIV-1 protease structures and elastic network modes. Structure. 2008;16: 321–30. 10.1016/j.str.2007.12.011 18275822 PMC2350220

[80] Meireles L, Gur M, Bakan A, Bahar I. Pre-existing soft modes of motion uniquely defined by native contact topology facilitate ligand binding to proteins. Protein Sci. 2011;20: 1645–58. 10.1002/pro.711 21826755 PMC3218357

[81] Ichiye T, Karplus M. Collective motions in proteins: a covariance analysis of atomic fluctuations in molecular dynamics and normal mode simulations. Proteins. 1991;11: 205–217. 10.1002/prot.340110305 1749773

[82] Kester WR, Matthews BW. Crystallographic study of the binding of dipeptide inhibitors to thermolysin: implications for the mechanism of catalysis. Biochemistry. 1977;16: 2506–2516. 861218 10.1021/bi00630a030

[83] Hausrath AC, Matthews BW. Thermolysin in the absence of substrate has an open conformation. Acta Crystallogr Sect D Biol Crystallogr. International Union of Crystallography; 2002;58: 1002–1007. 10.1107/S090744490200584X 12037302

[84] Holland DR, Tronrud DE, Pley HW, Flaherty KM, Stark W, Jansonius JN, et al Structural comparison suggests that thermolysin and related neutral proteases undergo hinge-bending motion during catalysis. Biochemistry. 1992;31: 11310–6. 1445869 10.1021/bi00161a008

[85] Van Aalten D, Amadei A, Linssen a B, Eijsink V, Vriend G, Berendsen HJC. The essential dynamics of thermolysin: Confirmation of the hinge-bending motion and comparison of simulations in vacuum and water. Proteins Struct Funct Genet. 1995;22: 45–54. 10.1002/prot.340220107 7675786

[86] Amadei a, Linssen a B, Berendsen HJ. Essential dynamics of proteins. Proteins. 1993;17: 412–25. 10.1002/prot.340170408 8108382

[87] Daidone I, Amadei A. Essential dynamics: foundation and applications. Wiley Interdiscip Rev Comput Mol Sci. 2012;2: 762–770. 10.1002/wcms.1099

[88] Ermakova E, Kurbanov R. Effect of ligand binding on the dynamics of trypsin. Comparison of different approaches. J Mol Graph Model. Elsevier Inc.; 2014;49: 99–109. 10.1016/j.jmgm.2014.02.001

[89] Skjaerven L, Martinez A, Reuter N. Principal component and normal mode analysis of proteins; a quantitative comparison using the GroEL subunit. Proteins Struct Funct Bioinforma. 2011;79: 232–243. 10.1002/prot.22875 21058295

[90] Bakan A, Bahar I. Computational Generation inhibitor-Bound Conformers of P38 Map Kinase and Comparison with Experiments. Pacific Symp Biocomput. 2011; 181–192. 10.1142/9789814335058_0020 PMC4782186 21121046

[91] Fuglebakk E, Echave J, Reuter N. Measuring and comparing structural fluctuation patterns in large protein datasets. Bioinformatics. 2012;28: 2431–40. 10.1093/bioinformatics/bts445 22796957

[92] Bahar I, Chennubhotla C, Tobi D. Intrinsic dynamics of enzymes in the unbound state and relation to allosteric regulation. Curr Opin Struct Biol. 2007;17: 633–640. 10.1016/j.sbi.2007.09.011 18024008 PMC2197162

[93] Eyal E, Yang L-W, Bahar I. Anisotropic network model: systematic evaluation and a new web interface. Bioinformatics. 2006;22: 2619–27. 10.1093/bioinformatics/btl448 16928735

[94] Fuglebakk E, Reuter N, Hinsen K. Evaluation of Protein Elastic Network Models Based on an Analysis of Collective Motions. J Chem Theory Comput. 2013;9: 5618–5628. 10.1021/ct400399x 26592296

[95] Rueda M, Chacón P, Orozco M. Thorough Validation of Protein Normal Mode Analysis: A Comparative Study with Essential Dynamics. Structure. 2007;15: 565–575. 10.1016/j.str.2007.03.013 17502102

[96] Riccardi D, Cui Q, Phillips GN. Evaluating elastic network models of crystalline biological molecules with temperature factors, correlated motions, and diffuse X-ray scattering. Biophys J. Biophysical Society; 2010;99: 2616–2625. 10.1016/j.bpj.2010.08.013 PMC2955396 20959103

[97] Romo TD, Grossfield A. Validating and improving elastic network models with molecular dynamics simulations. Proteins Struct Funct Bioinforma. 2011;79: 23–34. 10.1002/prot.22855 20872850

[98] Leioatts N, Romo TD, Grossfield A. Elastic network models are robust to variations in formalism. J Chem Theory Comput. 2012;8: 2424–2434. 10.1021/ct3000316 22924033 PMC3424003

[99] Kundu S, Melton JS, Sorensen DC, Phillips GN. Dynamics of proteins in crystals: comparison of experiment with simple models. Biophys J. Elsevier; 2002;83: 723–732. 10.1016/S0006-3495(02)75203-X PMC1302181 12124259

[100] Yang LW, Eyal E, Chennubhotla C, Jee J, Gronenborn AM, Bahar I. Insights into Equilibrium Dynamics of Proteins from Comparison of NMR and X-Ray Data with Computational Predictions. Structure. 2007;15: 741–749. 10.1016/j.str.2007.04.014 17562320 PMC2760440

[101] Gur M, Zomot E, Bahar I. Global motions exhibited by proteins in micro- to milliseconds simulations concur with anisotropic network model predictions. J Chem Phys. 2013;139: 121912 10.1063/1.4816375 24089724 PMC3739829

[102] Yang L-W, Bahar I. Coupling between catalytic site and collective dynamics: a requirement for mechanochemical activity of enzymes. Structure. 2005;13: 893–904. 10.1016/j.str.2005.03.015 15939021 PMC1489920

[103] Dutta A, Bahar I. Metal-binding sites are designed to achieve optimal mechanical and signaling properties. Structure. Elsevier Ltd; 2010;18: 1140–8. 10.1016/j.str.2010.06.013 PMC2937013 20826340

[104] Bakan A, Meireles LM, Bahar I. ProDy: protein dynamics inferred from theory and experiments. Bioinformatics. 2011;27: 1575–7. 10.1093/bioinformatics/btr168 21471012 PMC3102222

[105] Bahar I, Lezon TR, Bakan A, Shrivastava IH. Normal mode analysis of biomolecular structures: functional mechanisms of membrane proteins. Chem Rev. 2010;110: 1463–97. 10.1021/cr900095e 19785456 PMC2836427

[106] Atilgan AR, Durell SR, Jernigan RL, Demirel MC, Keskin O, Bahar I. Anisotropy of Fluctuation Dynamics of Proteins with an Elastic Network Model. 2001;80. 10.1016/S0006-3495(01)76033-X PMC1301252 11159421

[107] Brown CK, Madauss K, Lian W, Beck MR, Tolbert WD, Rodgers DW. Structure of neurolysin reveals a deep channel that limits substrate access. Proc Natl Acad Sci U S A. 2001;98: 3127–32. 10.1073/pnas.051633198 11248043 PMC30618

[108] Arndt JW, Hao B, Ramakrishnan V, Cheng T, Chan SI, Chan MK. Crystal structure of a novel carboxypeptidase from the hyperthermophilic archaeon Pyrococcus furiosus. Structure. 2002;10: 215–24. 11839307 10.1016/s0969-2126(02)00698-6

[109] Natesh R, Schwager S, Sturrock E, Acharya K. Crystal structure of the human angiotensin-converting enzyme—lisinopril complex. Nature. 2003;421: 551–554. 12540854 10.1038/nature01370

[110] Lee MM, Isaza CE, White JD, Chen RP-Y, Liang GF-C, He HT-F, et al Insight into the substrate length restriction of M32 carboxypeptidases: characterization of two distinct subfamilies. Proteins. 2009;77: 647–57. 10.1002/prot.22478 19544567

[111] Watermeyer JM, Sewell BT, Schwager SL, Natesh R, Corradi HR, Acharya KR, et al Structure of testis ACE glycosylation mutants and evidence for conserved domain movement. Biochemistry. 2006;45: 12654–63. 10.1021/bi061146z 17042482 PMC1892614

[112] Comellas-Bigler M, Lang R, Bode W, Maskos K. Crystal structure of the E. coli dipeptidyl carboxypeptidase Dcp: further indication of a ligand-dependent hinge movement mechanism. J Mol Biol. 2005;349: 99–112. 10.1016/j.jmb.2005.03.016 15876371

[113] Etges R, Bouvier J, Bordier C. The major surface protein of Leishmania promastigotes is a protease. J Biol Chem. 1986;261: 9098–101. 3522584

[114] Schlagenhauf E, Etges R, Metcalf P. The crystal structure of the Leishmania major surface proteinase leishmanolysin (gp63). Structure. 1998;6: 1035–46. 9739094 10.1016/s0969-2126(98)00104-x

[115] Bianchini G, Bocedi A, Ascenzi P, Gavuzzo E, Mazza F, Aschi M. Molecular dynamics simulation of Leishmania major surface metalloprotease GP63 (leishmanolysin). Proteins Struct Funct Bioinforma. 2006;64: 385–390. 10.1002/prot.21009 16708363

