# Supplementary material for: Comparison of the Internal Dynamics of Metalloproteases Provides New Insights on Their Function and Evolution
Source: PLoS One. 2015 Sep 23;10(9):e0138118. doi: 10.1371/journal.pone.0138118 (PMC4580569; doi:10.1371/journal.pone.0138118)
Supplement: S1 File — (DOCX) [file pone.0138118.s001.docx]

# Supporting Information – S1

Table A - Thermolysin structures (Uniprot ID: P00800) retrieved from the PDB. Unbound structures in bold. Reference structure for PC, NM and MD simulations in bold underlined.

| 1FJ3 | 1FJO | 1FJQ | 1FJT | 1FJU | 1FJV | 1FJW | 1GXW | 1HYT | 1KEI | 1KJO | 1KJP | 1KKK | 1KL6 | 1KR6 | 1KRO | 1KS7 | 1KTO | **1L3F** |
| --- | --- | --- | --- | --- | --- | --- | --- | --- | --- | --- | --- | --- | --- | --- | --- | --- | --- | --- |
| 1LNA | 1LNB | 1LNC | 1LND | 1LNE | 1LNF | 1OS0 | 1PE5 | 1PE7 | 1PE8 | 1QF0 | 1QF1 | 1QF2 | 1THL | 1TLI | 1TLP | 1TLX | 1TMN | 1Y3G |
| 1Z9G | 1ZDP | 2A7G | 2G4Z | 2TLI | 2TLX | 2TMN | 2WHZ | 2WI0 | 3DNZ | 3DO0 | 3DO1 | 3DO2 | 3EIM | 3F28 | 3F2P | 3FB0 | 3FBO | 3FCQ |
| 3FGD | 3FLF | 3FOR | 3FV4 | 3FVP | 3FXP | 3FXS | 3LS7 | 3MS3 | 3MSA | 3MSF | 3MSN | 3N21 | 3NN7 | 3P7P | 3P7Q | 3P7R | 3P7S | 3P7T |
| 3P7U | 3P7V | 3P7W | 3QGO | 3QH1 | 3QH5 | 3SSB | **3T2H** | **3T2I** | **3T2J** | 3T73 | 3T74 | 3T87 | 3T8C | 3T8D | 3T8F | 3T8G | 3T8H | 3TLI |
| 3TMN | 3ZI6 | 4D91 | 4D9W | 4H57 | 4TLI | 4TLN | 4TMN | 5TLI | 5TLN | 5TMN | 6TLI | 6TMN | 7TLI | 7TLN | 8TLI | 8TLN |  |  |

Structures were obtained using the Prody software (as of 09/2013). Bold: unbound crystal structure used for ANM generation. Underlined: unbound crystal structures obtained in the presence of cryoprotectors.

Table B - List of Z-scores and P-values obtained for the alignments of MP representative structures.

| Pair | Z-Score | P-value | Gluzincin | Mixed | Metzincin |
| --- | --- | --- | --- | --- | --- |
| M3-M27 | 0.9 | 6.18E-02 | x |  |  |
| M32-M27 | 1.2 | 9.37E-04 | x |  |  |
| M32-M10(B) | 1.3 | 1.29E-02 |  | x |  |
| M32-M8 | 1.3 | 2.55E-04 |  | x |  |
| M4-M10(B) | 1.3 | 3.35E-02 |  | x |  |
| M27-M10(A) | 1.5 | 4.55E-01 |  | x |  |
| M32-M12(B) | 1.9 | 2.12E-01 |  | x |  |
| M3-M35 | 1.9 | 4.87E-01 |  | x |  |
| M27-M10(B) | 1.9 | 5.43E-02 |  | x |  |
| M27-M8 | 1.9 | 1.47E-03 |  | x |  |
| M4-M27 | 2 | 2.32E-02 | x |  |  |
| M32-M10(A) | 2.1 | 4.54E-01 |  | x |  |
| M27-M7 | 2.1 | 4.46E-01 |  | x |  |
| M4-M12(B) | 2.1 | 8.04E-02 |  | x |  |
| M27-M12(B) | 2.2 | 1.89E-01 |  | x |  |
| M32-M35 | 2.2 | 3.39E-01 |  | x |  |
| M32-M12(A) | 2.3 | 1.27E-01 |  | x |  |
| M27-M35 | 2.3 | 1.91E-01 |  | x |  |
| M2-M12(B) | 2.3 | 4.14E-01 |  | x |  |
| M27-M12(A) | 2.3 | 1.81E-01 |  | x |  |
| M32-M7 | 2.4 | 3.90E-01 |  | x |  |
| M4-M8 | 2.6 | 9.08E-03 |  | x |  |
| M2-M27 | 2.7 | 4.98E-02 | x |  |  |
| M4-M10(A) | 2.7 | 1.48E-01 |  | x |  |
| M4-M12(A) | 2.7 | 4.06E-02 |  | x |  |
| M1-M27 | 2.8 | 5.12E-02 | x |  |  |
| M2-M8 | 2.8 | 1.22E-02 |  | x |  |
| M3-M12(A) | 2.8 | 4.03E-01 |  | x |  |
| M2-M10(B) | 2.9 | 8.12E-02 |  | x |  |
| M4-M35 | 2.9 | 4.48E-02 |  | x |  |
| M3-M12(B) | 3 | 4.46E-01 |  | x |  |
| M1-M12(A) | 3.1 | 3.79E-01 |  | x |  |
| M35-M8 | 3.1 | 2.97E-01 |  |  | x |
| M35-M12(A) | 3.2 | 1.48E-02 |  |  | x |
| M3-M10(B) | 3.2 | 9.09E-02 |  | x |  |
| M2-M35 | 3.2 | 4.57E-01 |  | x |  |
| M2-M12(A) | 3.2 | 3.77E-01 |  | x |  |
| M4-M7 | 3.3 | 3.24E-01 |  | x |  |
| M3-M8 | 3.4 | 2.12E-02 |  | x |  |
| M2-M10(A) | 3.5 | 5.27E-01 |  | x |  |
| M35-M10(B) | 3.7 | 2.80E-01 |  |  | x |
| M1-M12(B) | 3.7 | 4.11E-01 |  | x |  |
| M3-M7 | 3.7 | 5.29E-01 |  | x |  |
| M35-M10(A) | 3.9 | 3.62E-02 |  |  | x |
| M3-M10(A) | 3.9 | 5.32E-01 |  | x |  |
| M1-M10(B) | 4 | 1.52E-02 |  | x |  |
| M35-M12(B) | 4 | 1.00E-02 |  |  | x |
| M2-M7 | 4.1 | 5.29E-01 |  | x |  |
| M1-M35 | 4.2 | 3.91E-01 |  | x |  |
| M7-M35 | 4.2 | 3.06E-02 |  |  | x |
| M1-M10(A) | 4.2 | 4.98E-01 |  | x |  |
| M1-M7 | 4.3 | 5.17E-01 |  | x |  |
| M1-M8 | 4.4 | 5.52E-03 |  | x |  |
| M1-M2 | 4.8 | 1.27E-02 | x |  |  |
| M1-M3 | 5.4 | 7.00E-03 | x |  |  |
| M4-M32 | 5.9 | 1.77E-02 | x |  |  |
| M8-M12(A) | 6.1 | 1.68E-01 |  |  | x |
| M4-M3 | 6.5 | 9.02E-02 | x |  |  |
| M1-M32 | 6.6 | 9.06E-03 | x |  |  |
| M12(A)-M12(B) | 6.9 | 2.12E-02 |  |  | x |
| M4-M2 | 7.2 | 1.33E-01 | x |  |  |
| M8-M12(B) | 7.3 | 2.01E-01 |  |  | x |
| M8-M10(B) | 7.5 | 3.30E-03 |  |  | x |
| M10(B)-M12(B) | 7.7 | 3.07E-01 |  |  | x |
| M7-M8 | 7.8 | 4.67E-01 |  |  | x |
| M8-M10(A) | 8.6 | 3.84E-01 |  |  | x |
| M10(B)-M12(A) | 8.7 | 1.61E-01 |  |  | x |
| M7-M12(B) | 9.8 | 2.34E-02 |  |  | x |
| M12(A)-M10(A) | 10.8 | 1.65E-01 |  |  | x |
| M12(B)-M10(A) | 11.1 | 2.50E-02 |  |  | x |
| M7-M12(A) | 11.3 | 8.75E-02 |  |  | x |
| M7-M10(B) | 11.3 | 4.70E-01 |  |  | x |
| M4-M1 | 11.4 | 8.13E-02 | x |  |  |
| M7-M10(A) | 12.7 | 1.20E-01 |  |  | x |
| M10(B)-M10(A) | 13.8 | 3.18E-01 |  |  | x |
| M3-M2 | 19.2 | 4.78E-04 | x |  |  |
| M3-M32 | 19.7 | 6.94E-06 | x |  |  |
| M32-M2 | 21.4 | 2.97E-05 | x |  |  |
