# Supplementary material for: Comparison of the Internal Dynamics of Metalloproteases Provides New Insights on Their Function and Evolution
Source: PLoS One. 2015 Sep 23;10(9):e0138118. doi: 10.1371/journal.pone.0138118 (PMC4580569; doi:10.1371/journal.pone.0138118)
Supplement: S2 File — Neurolysin K148 and Carboxypeptidase Pfu R92 in bond representation. Active site residues in colored surface representations (Neurolysin: H474, E 475, H478 and E503; Carboxypeptidase Pfu H269, E270, H273 and E299) (Fig B). (DOCX) [file pone.0138118.s002.docx]

# Supporting Information – S2

Figure A: Root Mean Square Deviations of residue C^α^ obtained for Sim1 and Sim2.

Figure B: Dynamics-based alignment of Neurolysin (Blue, PDB ID: 1I1I) and Carboxypeptidase Pfu (red, PDB ID: 1KA4). Neurolysin K148 and Carboxypeptidase Pfu R92 in bond representation. Active site residues in colored surface representations (Neurolysin: H474, E 475, H478 and E503; Carboxypeptidase Pfu H269, E270, H273 and E299).
